# Theranostic PSMA ligands with optimized backbones for intraoperative multimodal imaging and photodynamic therapy of prostate cancer

**DOI:** 10.1007/s00259-022-05685-0

**Published:** 2022-01-14

**Authors:** Yvonne H. W. Derks, Sanne A. M. van Lith, Helene I. V. Amatdjais-Groenen, Lieke W. M. Wouters, Annemarie Kip, Gerben M. Franssen, Peter Laverman, Dennis W. P. M. Löwik, Sandra Heskamp, Mark Rijpkema

**Affiliations:** 1 Department of Medical Imaging, Nuclear Medicine, Radboud University Medical Center, Radboud Institute for Molecular Life Sciences, Geert Grooteplein Zuid 10, 6525GA Nijmegen, The Netherlands; 2 Institute for Molecules and Materials, Organic Chemistry, Radboud University Nijmegen, Nijmegen, The Netherlands

**Keywords:** PSMA, Prostate cancer, Multimodal imaging, Intraoperative, Photodynamic therapy

## Abstract

**Introduction:**

The first generation ligands for prostate-specific membrane antigen (PSMA)–targeted radio- and fluorescence-guided surgery followed by adjuvant photodynamic therapy (PDT) have already shown the potential of this approach. Here, we developed three new photosensitizer-based dual-labeled PSMA ligands by crucial modification of existing PSMA ligand backbone structures (PSMA-1007/PSMA-617) for multimodal imaging and targeted PDT of PCa.

**Methods:**

Various new PSMA ligands were synthesized using solid-phase chemistry and provided with a DOTA chelator for ^111^In labeling and the fluorophore/photosensitizer IRDye700DX. The performance of three new dual-labeled ligands was compared with a previously published first-generation ligand (PSMA-N064) and a control ligand with an incomplete PSMA-binding motif. PSMA specificity, affinity, and PDT efficacy of these ligands were determined in LS174T-PSMA cells and control LS174T wildtype cells. Tumor targeting properties were evaluated in BALB/c nude mice with subcutaneous LS174T-PSMA and LS174T wildtype tumors using µSPECT/CT imaging, fluorescence imaging, and biodistribution studies after dissection.

**Results:**

In order to synthesize the new dual-labeled ligands, we modified the PSMA peptide linker by substitution of a glutamic acid into a lysine residue, providing a handle for conjugation of multiple functional moieties. Ligand optimization showed that the new backbone structure leads to high-affinity PSMA ligands (all IC_50_ < 50 nM). Moreover, ligand-mediated PDT led to a PSMA-specific decrease in cell viability in vitro (*P* < 0.001). Linker modification significantly improved tumor targeting compared to the previously developed PSMA-N064 ligand (≥ 20 ± 3%ID/g vs 14 ± 2%ID/g, *P* < 0.01) and enabled specific visualization of PMSA-positive tumors using both radionuclide and fluorescence imaging in mice.

**Conclusion:**

The new high-affinity dual-labeled PSMA-targeting ligands with optimized backbone compositions showed increased tumor targeting and enabled multimodal image-guided PCa surgery combined with targeted photodynamic therapy.

**Supplementary Information:**

The online version contains supplementary material available at 10.1007/s00259-022-05685-0.

## Introduction

Despite recent advances in imaging, staging, and therapy, prostate cancer (PCa) remains a significant health problem with a substantial morbidity and mortality [1]. First-line PCa treatment often consists of the surgical removal of the prostate [2]. Unfortunately, the narrow tumor resections performed to prevent comorbidities lead to positive surgical margins in 5–30% of patients, which can even increase up to 65% of patients in case of extra-prostatic extension of the tumor (pT3-pT4) [3–5]. Moreover, metastatic lymph nodes embedded in highly vascularized abdominal lipid tissue can easily be missed by the surgeon, leading to biochemical recurrences in up to 35% of these patients [6, 7].

The challenges mentioned above stress the importance of improved intraoperative visualization of tumor margins and adjuvant ablative procedures for the primary tumor and improved tumor detection in (metastatic) lymph nodes. A promising strategy to achieve these goals is combined radio- and fluorescence-guided surgery followed by intraoperative photodynamic therapy (PDT) [8–10]. PDT is a method to induce cellular damage through administration and subsequent selective activation of a photosensitizer. Excitation of the photosensitizer induces fluorescence for intraoperative fluorescence imaging [8, 9], but it also leads to the production of highly toxic singlet oxygen (^1^O_2_) and reactive oxygen species (ROS) [11–13]. ROS and ^1^O_2_ can cause immunogenic, necrotic, and apoptotic cell death [11, 14–16].

A highly suitable target for imaging and therapy in PCa is the prostate-specific membrane antigen (PSMA) [17, 18]. In the past decade, characterization of the active substrate recognition site of PSMA has allowed for the development of numerous highly specific small-molecule PSMA-targeting ligands [19–22]. Previously, our group developed a first-generation photosensitizer-based dual-labeled PSMA ligand for intra-operative imaging and therapy of PCa called PSMA-N064, which showed the potential of this approach in PSMA-positive xenografts [23]. Nonetheless, achieving the highest possible tumor uptake is essential for fluorescence imaging and PDT, warranting further ligand optimization.

Well-known high-affinity PSMA targeting tracers with excellent tumor uptake that are currently used in clinical trials include PSMA-617 and PSMA-1007 [19–21]. These ligands precisely fit both the active site and the entrance funnel of PSMA [19, 20, 24, 25]. Since PSMA-617 and PSMA-1007 are not dual-labeled and lack a photosensitizer, they are not suited for multimodal intraoperative imaging and PDT of PCa. However, backbone modification of these high-affinity ligands to provide a handle for multiple functional moieties could lead to dual-labeled ligands while preserving excellent tumor uptake. Therefore, we made a crucial modification to the backbone of PSMA-1007 by the incorporation of a lysine side residue. Based on the crystal structure of PSMA-1007 in the active site of PSMA (Supplementary Fig. 1), the side chain of this lysine residue is oriented towards the exterior of PSMA, providing ample space for (multiple) functional elements [20, 25].

**Fig. 1:**
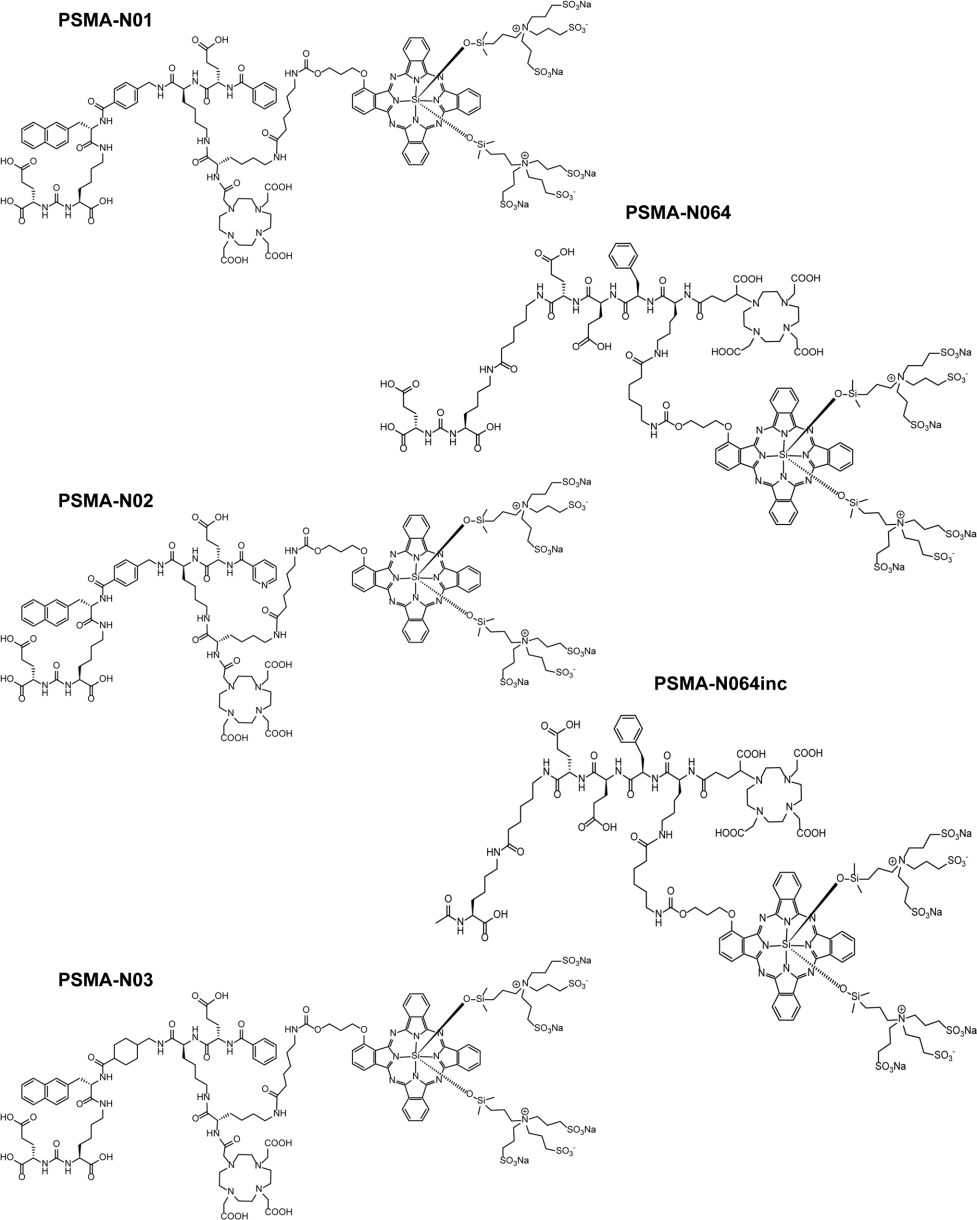
Structures of DOTA(GA)-IRDye700DX-PSMA ligands with different backbone compositions

Using these new backbones, we synthesized three dual-labeled PSMA ligands consisting of both the photosensitizer/fluorophore IRDye700DX and a DOTA chelator for indium-111 (^111^In) labeling (Fig. 1). Affinity, PSMA-targeted PDT potential, and tumor uptake of the new dual-labeled ligands were determined using PSMA-expressing tumor cells and PSMA-positive xenograft models. Moreover, we directly compared ligand performance with our previously published first-generation ligand (PSMA-N064) and a control ligand with an incomplete PSMA-binding motif (PSMA-N064inc) [23].

## Materials and methods

### Synthesis of dual-labeled ligands

The new PSMA-binding ligands (PSMA-N01, PSMA-N02, and PSMA-N03) were synthesized using solid-phase chemistry. After cleavage from the resin, the ligands were conjugated with IRDye700DX in solution using N-hydroxysuccinimide chemistry. Full synthetic procedures and results can be found in the supplementary data (Page 1–4, Supplementary Fig. 2). Regarding PSMA-N064, we previously published a detailed description of the synthetic procedures and chemical analyses (reverse-phase high-performance liquid chromatography (RP-HPLC), matrix-assisted laser desorption ionization time-of-flight (MALDI-ToF)) [23]. As a control, a ligand similar to PSMA-N064 was included that is lacking the glutamic acid in the PSMA-binding motif, referred to as PSMA-N064-incomplete (PSMA-N064inc).

### Cell culture

LS174T cell line was acquired from the American Type Culture Collection. LS174T colon carcinoma cells were stably transfected DNA encoding for human PSMA using the plasmid pcDNA3.1-hPSMA as described before [3]. Cells were cultured in RPMI 1640 medium supplemented with 10% FCS and 2-mM glutamine (5% CO_2_, 37 °C). LS174T-PSMA cells were cultured in the presence of 0.3 mg/ml G418 geneticin as well.

### Radiolabeling and RP-HPLC

Peptides were labeled under metal-free conditions with ^111^InCl_3_ (Curium) in 0.5 M 2-(N-morpholino)ethanesulfonic acid (MES) buffer (pH 5.5, twice volume of ^111^InCl_3_) or sodium acetate buffer (NaOAc in 0.04 M acetic acid solution, pH 4.5). Labeling was performed at 45 °C for 10 min [26]. To chelate unincorporated ^111^InCl_3_, ethylenediamine-tetraacetic acid (EDTA, 50 mM) was added to a final concentration of 5 mM after the incubation.

Radiochemical yield (RCY) was determined by instant thin-layer chromatography (ITLC) using silica gel-coated paper (Agilent Technologies) and 0.1 M ammonium acetate containing 0.1 M EDTA, pH 5.5, as the mobile phase. In addition, RCY was determined using RP-HPLC on an Agilent 1200 system (Agilent Technologies) with an in-line radiodetector (Elysisa-Raytest). A reversed-phase C18 column (5 µm, 4.6 × 250 mm; HiChrom) was used at a flow rate of 1 ml/min. We used the following buffer system: buffer A, triethylammonium acetate (TEAA, 10 mM, pH 7); buffer B, 100% methanol; and a gradient of 97 to 0% buffer A (35 min). Peptides were purified by a Sep-Pak C18 light cartridge (Waters) and eluted from the cartridge with 50% ethanol in water.

### *In vitro* internalization assay

The binding and internalization characteristics of the ligands were determined using LS174T-PSMA cells. Cells were cultured to confluency in 6-well plates followed by incubation with 50,000 counts per minute of ^111^In-labeled ligand (0.1–0.25 pmol/well) in 1-ml RPMI + 0.5% BSA (37 °C, 2 h). Non-specific binding was determined by coincubation with 2(phosphonomethyl)-pentane-1,5-dioic acid (2-PMPA, 21.57 µM). PSMA-specific binding was defined as total binding minus the non-specific binding. To retrieve the membrane-bound fraction, cells were washed twice with PBS and incubated for 10 min at 0 °C with acid buffer (154-mM NaCl, 0.1 M acetic acid, pH 2.6). After incubation, the membrane-bound fraction was collected. Then, cells were washed and lysed with 0.1 M NaOH, and the cell lysate (intracellular activity) was collected. Intracellular and membrane-bound activity fractions were measured in a gamma-counter (2480 WIZARD^2^ Automatic Gamma Counter, PerkinElmer) [3, 27].

### *In vitro* targeted PDT assays

LS174T wildtype (LS174T-WT) and LS174T-PSMA cells were cultured to confluency in 48-well plates. Cells were incubated (2 h, 5% CO_2_, 37 °C) with 30-nM PSMA ligand in binding buffer (RPMI 1640 + 0.5% BSA) in triplicate. After washing with PBS, a 0.5-ml binding buffer was added to each well, and cells were irradiated with a NIR light-emitting diode (690 ± 20 nm) [28]. The typical forward voltage was 2.6 V creating a power output of 490 mW using 126 individual LED bulbs to ensure homogenous illumination of the area of interest predefined as 5 × 3 cm. The cells were irradiated at NIR radiant exposures of 100 J/cm^2^ (450 mW/cm^2^) and subsequently incubated for 1 h at 37 °C. Cells that only received the PSMA ligand, only the NIR light, or neither the ligand nor the light were included as controls. Cytotoxic effects of PDT with PSMA ligands were determined with a CellTiter-Glo™ assay (Promega Benelux) according to the manufacturer’s instructions. The binding buffer was replaced with 100-µl fresh binding buffer and 100-µl CellTiter-Glo® 2.0 Assay. Plates were shaken (2 min) and incubated for 10 min at room temperature. Next, luminescence was measured in a plate reader (Tecan Infinite® 200 PRO) to determine the metabolic activity of the cells.

### Animal tumor model

All animal experiments were approved by the institutional Animal Welfare Committee of the Radboud University Medical Center and were conducted in accordance to the guidelines of the Revised Dutch Act on Animal Experimentation. Animal experiments were performed in 8–10 weeks old male BALB/c nude mice (Janvier). The mice were housed in individually ventilated cages (Blue line IVC, 3–5 mice per cage), under standard non-sterile conditions with cage enrichment present. There was free access to chlorophyll-free animal chow (Sniff Voer) and water. Mice were subcutaneously inoculated with 3.0 × 10^6^ LS174T-PSMA cells in the right flank and 1.5 × 10^6^ LS174T WT cells in the left flank (diluted in 200-µl RPMI 1640 medium). When xenografts were approximately 0.5 cm^3^ (10–14 days after injection), mice were block-randomized into groups based on tumor size. The researchers were not blinded for the experimental groups.

### *In vivo *biodistribution, SPECT/CT imaging, and fluorescence imaging

Mice were intravenously injected with 0.3 nmol PSMA ligand, labeled with 10 MBq ^111^In (molar activity 33.3 MBq/nmol) in PBS + 0.5% (w/v) BSA. For the ex vivo biodistribution, five groups (one group for each ligand) of four mice were included. Two hours p.i., all mice were euthanized by CO_2_/O_2_-asphyxiation. For two mice of each group (2 mice/ligand), background-subtracted fluorescence images were acquired with the IVIS imaging system (Xenogen VivoVision IVIS Lumina II, PerkinElmer), with a 640-nm excitation filter and a Cy5.5 emission filter and an acquisition time of 10 s. Next, µSPECT/CT imaging was performed in the same two mice per group, with a 1.0-mm diameter pinhole mouse collimator tube (U-SPECT II, MILabs) [29]. Mice were scanned for 30 min followed by a CT scan for anatomical reference (spatial resolution 160 μm, 615 μA, 65 kV). MILabs reconstruction software was used to reconstruct the µSPECT/CT scans, via an ordered-subset expectation maximization algorithm, energy windows 154–188 keV and 220–270 keV, 3 iterations, 16 subsets, and voxel size of 0.75 mm. SPECT/CT maximum intensity projections (MIPs) were created using the Inveon Research Workplace software (Siemens Preclinical Solutions, version 4.1). NIR fluorescence images were analyzed using Living Image software (PerkinElmer, version 4.2). After imaging, relevant tissues were dissected, weighed, and measured for radioactivity in a gamma-counter (2480 WIZARD^2^ Automatic Gamma Counter, PerkinElmer). In addition, a blocking experiment with PSMA-N064 and PSMA-617 was performed. Full experimental procedures and results can be found in the supplementary data (Page 5, Supplementary Fig. 3).

### Statistical analysis

Graphpad Prism software (version 5.03) was used to perform statistical analyses. Results are presented as mean ± SD. Differences in in vitro PDT efficacy, affinity, and in vivo tumor and organ uptake were tested for significance using a one-way ANOVA with a Bonferroni’s multiple comparisons posttest. Differences were considered significant at *P* < 0.05, two-sided.

## Results

### Design and synthesis of the ligands

**Fig. 2:**
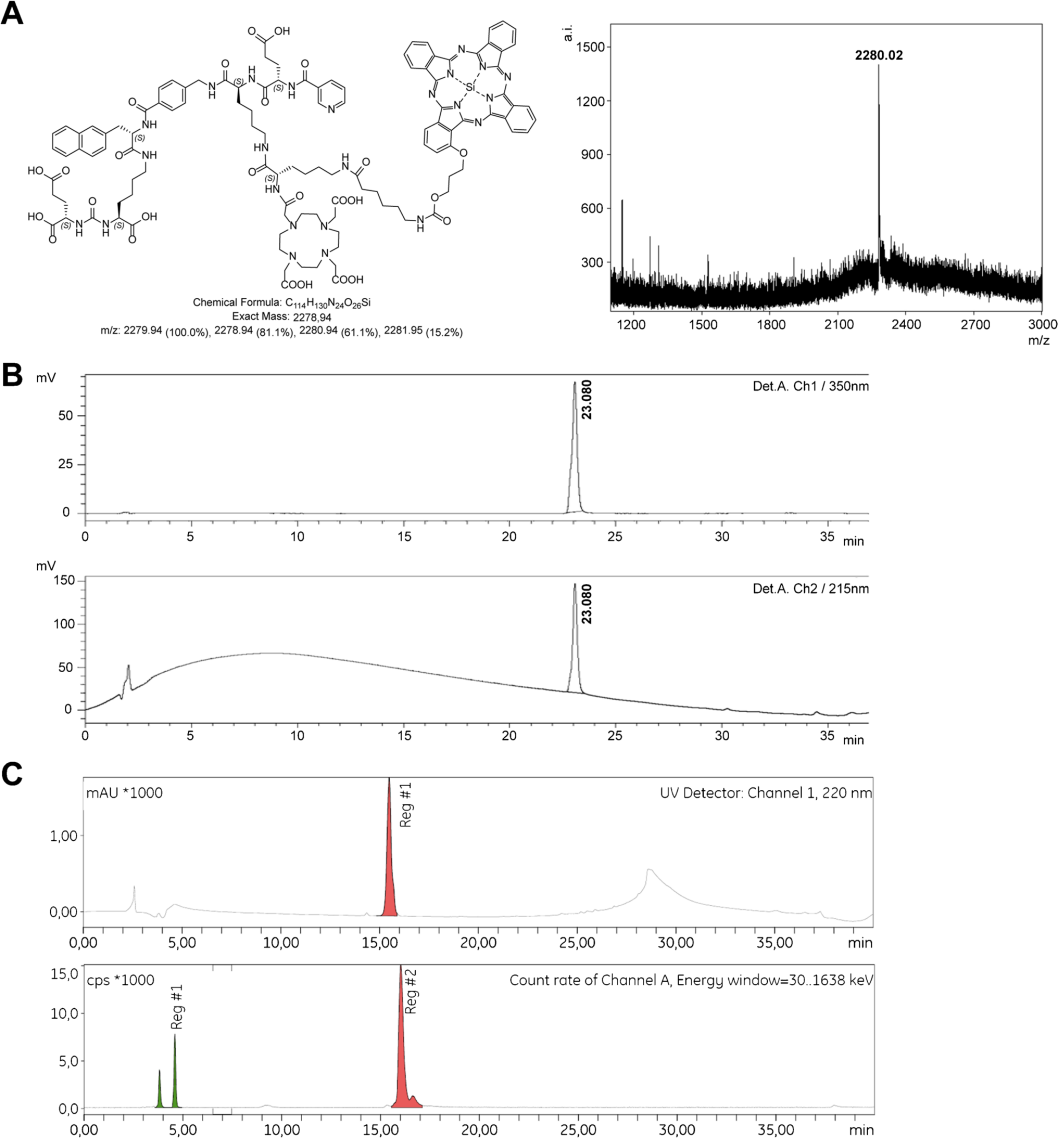
Chemical analysis and radiolabeling of PSMA-N02. **a** Mass spectrometry of non-radiolabeled PSMA-N02; MALDI-TOF spectrum using a α-cyano-4-hydroxycinnamic acid (HCCA) matrix. **b** Analytical RP-HPLC of non-radiolabeled PSMA-N02 with detectors at 350 nm (a low-range absorption peak of IRDye700DX) and 215 nm. **c** RP-HPLC of PSMA-N02 before and after labeling (specific activity 8 MBq/µg) in NaOAc buffer suitable for clinical translation. RP-HPLC before purification shows free ^111^In (green peaks) and [^111^In]In-DOTA-PSMA-N02 (red peak, RCY 80%)

We designed three glutamate-urea-lysine-based PSMA ligands with various backbones conjugated to DOTA and IRDye700DX (Fig. 1). The design of our ligands is based on high-affinity ligands PSMA-1007 and PSMA-617. This means that they consist of naphtylalanine, aminomethyl benzoic acid, aminomethyl cyclohexane, glutamic acid, benzoic acid, and nicotinic acid (not-fluorinated) groups. However, we introduced extra-functional groups to the linker by substitution of the most C-terminal glutamic acid of PSMA-1007 into a lysine residue (red circle, Supplementary Fig. 1). Next, an additional lysine residue was connected to the lysine ε-amine of the peptide linker. With this modification, we aimed to preserve the perfect fit of the ligands in PSMA and their high affinity towards PSMA while enabling dual-labeling of the ligands. The exact differences between the backbone structures of the three newly synthesized ligands are as follows: PSMA-N01 and PSMA-N02 are PSMA-1007-based and thus contain a 4-(aminomethyl)benzoic acid, whereas PSMA-N03 is PSMA-617-based and therefore contains a 4-(aminomethyl)cyclohexane-1-carboxylic acid. Moreover, PSMA-N02 was capped with a nicotinic acid instead of a benzoic acid on its N-terminus, which was hypothesized to form an extra-hydrogen bond with PSMA (Fig. 1). As a control, we included two previously developed dual-labeled ligands called PSMA-N064 and PSMA-N064inc that have a backbone partly based on PSMA-I&T (Fig. 1). Chemical analysis using MALDI-TOF and RP-HPLC confirmed the synthesis of all three ligands (PSMA-N01, -N02, -N03) as well as the two control ligands (PSMA-N064, PSMA-N064inc) (Supplementary Fig. 4). As an example, the chemical analysis of PSMA-N02 is depicted in Fig. 2.

**Fig. 3:**
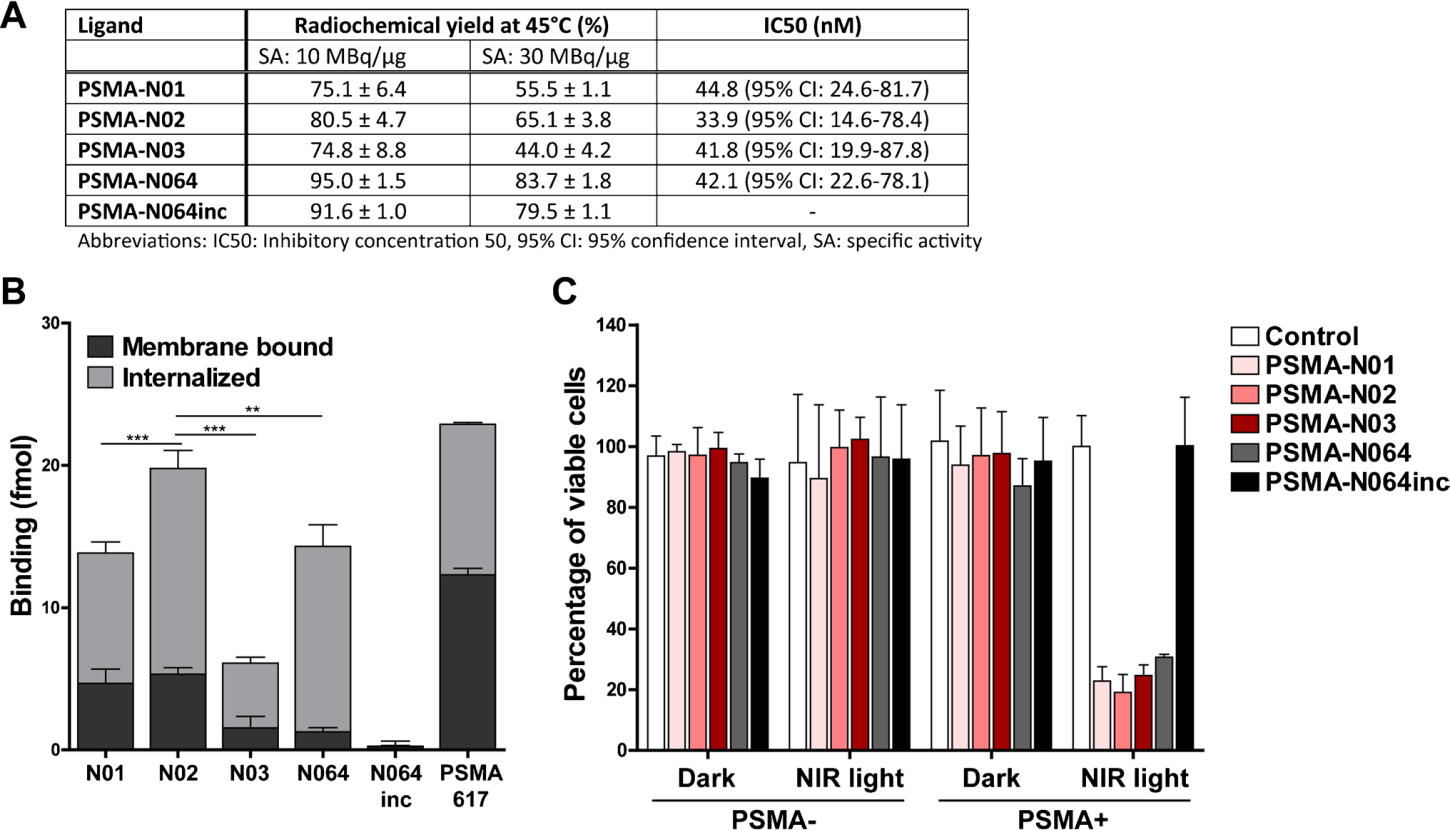
In vitro characterization of [^111^In]In-DOTA(GA)-IRDye700DX-PSMA ligands varying in their backbone composition. **a** Labeling efficiency of [^111^In]In-DOTA-PSMA-N01, -N02, -N03, [^111^In]In-DOTAGA-PSMA-N064 and -N064inc at a specific activity of either 10 MBq/µg or 30 MBq/µg (*n* = 2). IC_50_ values of ligands as determined in competitive binding assays using LS174T-PSMA cells. **b** Binding and internalization of [^111^In]In-DOTA-PSMA-N01, -N02, -N03, [^111^In]In-DOTAGA-PSMA-N064 and -N064inc in LS174T PSMA-positive cells. Membrane binding and internalization are corrected for non-specific binding and accumulation, as determined by co-incubation with an excess of 2-PMPA (50 µg). [^111^In]In-DOTA-PSMA-617 was added as a positive control. **c** PSMA-targeted PDT efficacy of ligands in vitro. Cell viability of LS174T-PSMA (PSMA +) and LS174T wildtype (PSMA-) cells following incubation with 30 nM of PSMA-N01, -N02, -N03, -N064, and -N064inc, after either a 100 J/cm^2^ (450 mW/cm^2^) radiant exposure or no light exposure (dark). Data is expressed as mean ± SD, ^**^P < 0.01, ^***^P < 0.001

We further verified the radiolabeling potential of these ligands at different specific activities (Fig. 3a). Labeling at a specific activity of 10 MBq/µg (10 min, 45 °C) led to a radiochemical yield (RCY) of 75% ± 6.4%, 81% ± 4.7%, and 75% ± 8.8% for DOTA-conjugated PSMA-N01, -N02, and -N03, respectively. In comparison, RCY of DOTAGA-conjugated PSMA-N064 and PSMA-N064inc exceeded 90%. RCY during labeling at 30 MBq/µg was 55% ± 1.1%, 65% ± 3.8%, and 44% ± 4.2% for PSMA-N01, -N02, and -N03 respectively. In contrast, at 30 MBq/µg RCY of PSMA-N064 remained high at 84% ± 1.8% (Fig. 3a). For in vitro and in vivo experiments, all ligands were purified using solid-phase extraction on SepPak C18 cartridges, leading to final radiochemical purities of more than 95%. As a step towards clinical translation, we also performed the radiolabeling of PSMA-N02 (specific activity 8 MBq/µg) with ^111^In in a NaOAc buffer suitable for clinical translation, resulting in a RCY of 80%. Figure 2c shows the HPLC profile before purification with free ^111^In in green and [^111^In]In-DOTA-PSMA-N02 in red.

### *In vitro *characterization of ligands

IC_50_ determination showed that all ligands had a similar IC_50_ in the low nanomolar range (Fig. 3a). Next, the PSMA-binding potential of the ligands was examined in an in vitro binding and internalization assay using PSMA-expressing LS174T cells, in which all ligands showed PSMA-specific binding (Fig. 3b). A direct comparison of the three ligands revealed that PSMA-N02 has the highest membrane-bound and internalized fraction (*P* < 0.01). As expected, we observed no binding and internalization upon incubation with control ligand PSMA-N064inc, signifying the specificity of the ligands. Next, we compared the in vitro targeted PDT effects between the three ligands and control ligands (Fig. 3c). When cells were incubated with 30-nM ligand and irradiated with 100 J/cm^2^, cell viabilities of 23% ± 5%, 19% ± 6%, and 25% ± 4% were observed for PSMA-N01, -N02, and -N03, respectively. The targeted PDT efficacy did not significantly differ between the three ligands and also did not differ from PSMA-N064 (30% ± 1%, *p* = 0.053). After incubation with 30-nM PSMA-N064inc, cell viability was not affected (100% ± 16%). Cell viability of controls, consisting of irradiated PSMA-negative LS174T-WT cells and non-irradiated LS174T-PSMA and LS174T-WT cells, was also not affected (cell viability range 87–102%).

### Backbone modifications influence tumor uptake of ligands

**Fig. 4:**
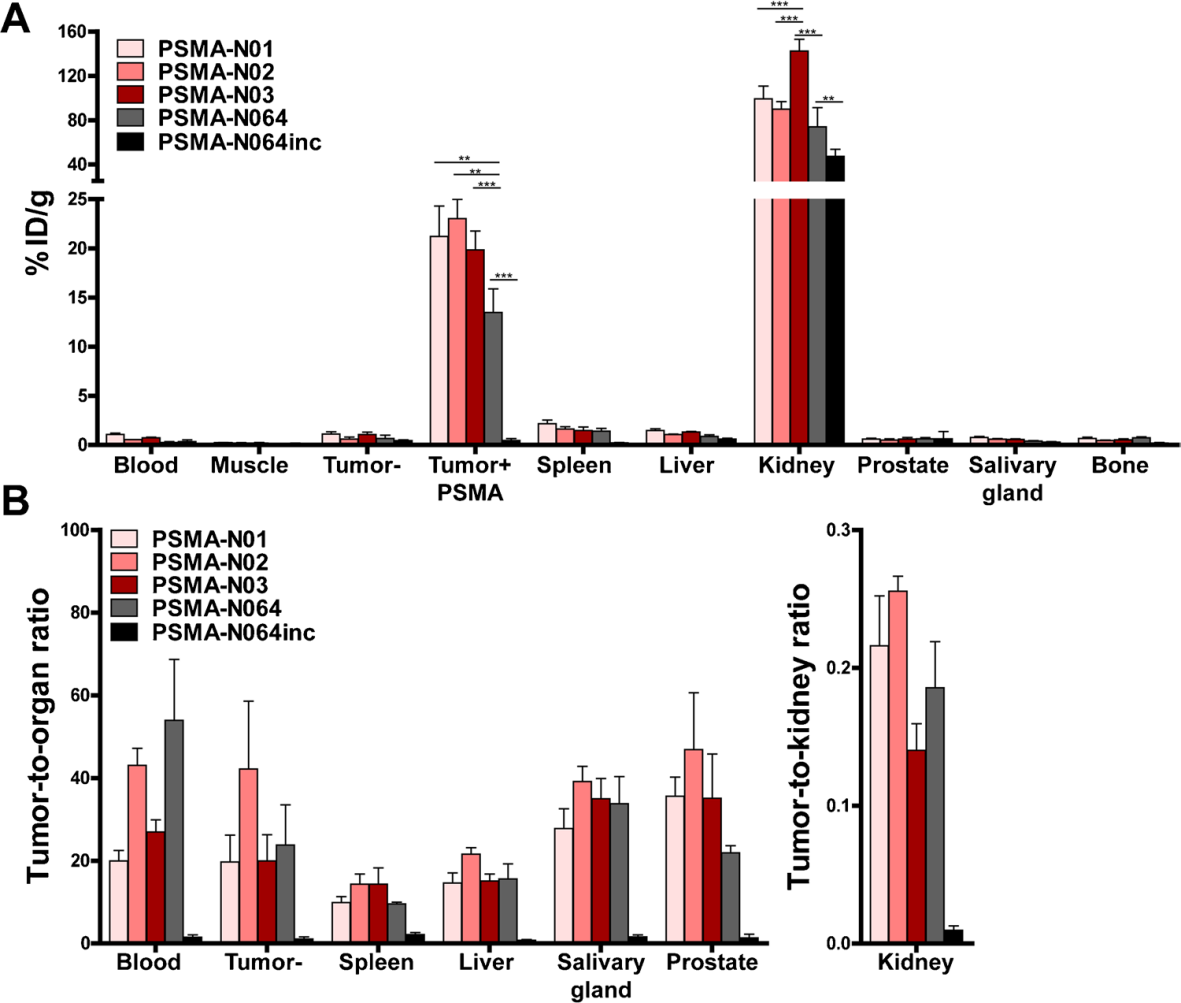
Backbone modifications in [^111^In]In-DOTA(GA)-IRDye700DX-PSMA ligands influence tumor uptake. Biodistribution as determined after dissection **a** and resulting tumor-to-organ ratios **b** of [^111^In]In-DOTA-PSMA-N01, -N02, -N03, and control ligands [^111^In]In-DOTAGA-PSMA-N064 and -N064inc (0.3 nmol, 10 MBq/mouse, 2 h p.i., *n* = 4/group). Biodistribution was determined in mice bearing subcutaneous LS174T-PSMA (labeled Tumor + PSMA) and LS174T wildtype (labeled Tumor-) xenografts. Data is expressed as %ID/g ± SD, ^**^P < 0.01, ^***^P < 0.001

To elucidate the importance of the backbone composition on ligand accumulation in PSMA-expressing tumors, we compared uptake of the three new dual-labeled ligands with the uptake of PSMA-N064, and control ligand PSMA-N064inc. All ligands showed uptake in LS174T PSMA-positive tumors, which was significantly higher compared with uptake in PSMA-negative tumors (*P* < 0.001) (Fig. 4a, Table S1). PSMA-N01, -N02, and -N03 showed a comparable uptake of 21 ± 3, 23 ± 2, and 20 ± 2%ID/g in the PSMA-positive tumor, respectively. The uptake of PSMA-N064 was significantly lower (14 ± 2%ID/g (*P* < 0.01) and the control ligand PSMA-N064inc showed minimal uptake of 0.5 ± 0.2%ID/g. For comparison, we also measured the uptake of PSMA-617 in our LS174T-PSMA tumor model, which was 19 ± 2%ID/g (Supplementary Fig. 3A, Table S2). In addition, we determined the specificity of PSMA-N064 in a blocking experiment (Supplementary Fig. 3B, Table S2). Blocking of PSMA-N064 with a 100 × excess of unlabeled PSMA-617 led to a decrease in PSMA-positive tumor uptake from 12 ± 2 to 1 ± 0.1%ID/g (*P* < 0.001).

We measured minimal uptake of our ligands in blood, spleen, liver salivary glands, and prostate, leading to high tumor-to-organ ratios for all three ligands (Fig. 4b, Table S1). Tumor-to-organ ratios were similar to those of PSMA-N064 and significantly higher compared with the control ligand PSMA-N064inc (*P* < 0.001). Ligand uptake in the excretory organ, the kidneys, was 90 ± 7%ID/g and 99 ± 12%ID/g and for PSMA-N01 and PSMA-N02, respectively (Fig. 4a). In comparison, kidney accumulation of PSMA-N03 was significantly higher (142 ± 11%ID/g, *P* < 0.001). Kidney accumulation of PSMA-N064 was 74 ± 17%ID/g, which was lower compared to PSMA-N01 (not significant), -N02 (not significant), and -N03 (*P* < 0.001). The control PSMA-N064inc ligand demonstrated a significantly lower kidney uptake of 47 ± 6%ID/g (*P* < 0.01), suggesting that kidney uptake in mice is partly PSMA-specific.

### Ligand-mediated multimodal imaging of PSMA-expressing tumors

**Fig. 5:**
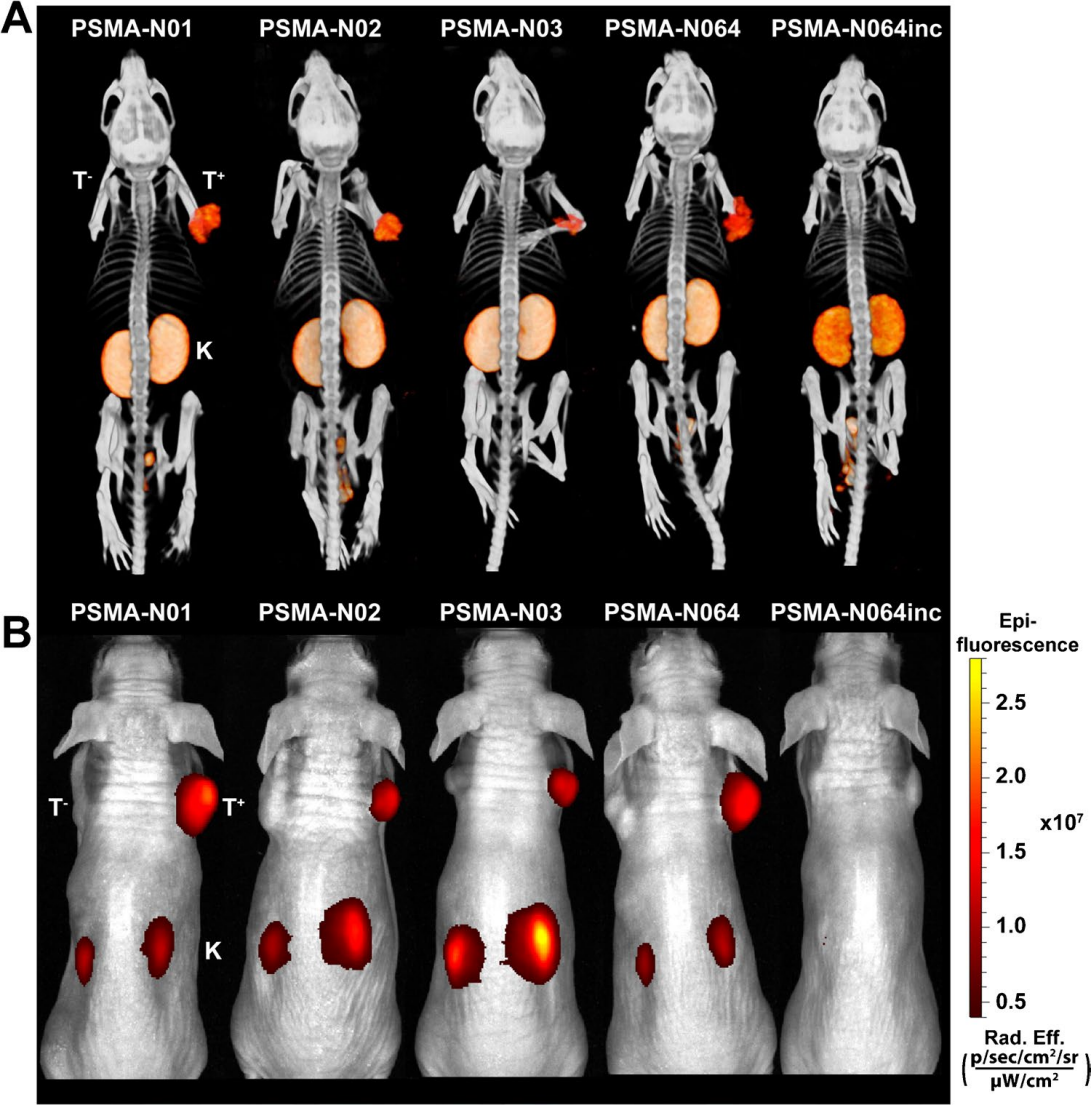
µSPECT/CT and fluorescence images of dual-labeled PSMA-ligands. Representative same scale µSPECT/CT scans **a** and fluorescence images **b** of mice with s.c. LS174T-PSMA (T^+^, right) and wildtype LS174T (T^−^, left) tumors after i.v. injection of [^111^In]In-DOTA(GA)-PSMA ligands (0.3 nmol, 10 MBq/mouse, 2 h p.i.). Abbreviations: T^+^; PSMA-positive tumor, T^−^; PSMA-negative tumor, K; kidney

To determine the imaging potential of our new ligands, we scanned two mice per group with a NIR fluorescence scanner and a μSPECT/CT scanner. Representative images of all ligands are shown in Fig. 5a and b. Using both imaging modalities, the subcutaneous LS174T PSMA-positive tumors (right flank) could be clearly visualized with all ligands, except for PSMA-N064inc. PSMA-negative LS174T WT tumors (left flank) demonstrated no visible ligand uptake. The images visualized high renal ligand accumulation in all mice, which was lowest for PSMA-N064inc in accordance with biodistribution results.

## Discussion

Local and metastatic relapses often occur following intended curative resection of PCa [2, 4, 5]. Radio- and fluorescence-guided surgery followed by adjuvant photodynamic therapy is a promising strategy that may assist the surgeon to achieve complete removal of tumor tissue while sparing surrounding healthy tissue. In our previous work and the current study, we developed and characterized dual-labeled PSMA-targeting ligands suited for this strategy [23]. These ligands allowed for highly specific tumor localization, visualization, and PDT in PSMA-expressing tumor cells and xenograft models.

Proper ligand design, including a backbone connecting the PSMA binding motif to one or multiple functional elements of the ligand, is highly important as it must enable high-affinity binding of the ligand to the active site of PSMA and result in favorable pharmacokinetic properties [30, 31]. Previously, we developed and characterized the PSMA-N064 ligand and its control PSMA-N064inc, demonstrating the proof-of-concept for dual-labeled PSMA-targeted imaging and PDT [23]. Nonetheless, we continued ligand development since achieving the highest possible tumor uptake of the ligands is essential, particularly for fluorescence imaging and PDT. For PDT, high absolute uptake in the tumor may lead to increased PDT effects, and may mean that less NIR exposure is needed to produce sufficient amounts of oxygen radicals, possibly leading to fewer side effects of the treatment [14–16].

With the aim to develop dual-labeled PSMA ligands that have the highest possible tumor uptake, we merged the chemical structure of PSMA-N064 with those of well-known high-affinity ligands PSMA-1007 and PSMA-617 [19, 20]. We incorporated a lysine residue in the peptide backbones, of which the side chain is postulated to point towards the exterior of PSMA. On this lysine, we attached an additional lysine residue to providing handles for conjugation of multiple functional moieties [25].

In vitro, the dual-labeled ligands with a DOTA chelator (PSMA-N01, -N02, and -N03) had a lower labeling efficiency compared with the DOTAGA-based PSMA-N064 and PSMA-N064inc (45 °C). Nonetheless, all ligands could be purified, leading to radiochemical purities > 95%. PSMA-N01, -N02, and -N03 all had a PSMA affinity in the nanomolar range (IC_50_ < 50 nM) and showed internalization percentages of 73–90% in the LS174T-PSMA cells (percentage of cell-associated ligand that was internalized). In a head-to-head comparison, [^111^In]In-DOTA-PSMA-617 demonstrated an IC_50_ of 52.7 nM and an internalization ratio of 46% [23]. The IC_50_ of ^18^F-PSMA-1007 reported in the literature is 4.2 ± 0.5 nM and the internalization ratio is 67% [20, 32], indicating that in vitro performance of our newly developed ligands is in a similar range to that of PSMA-617 and PSMA-1007.

In vivo, we demonstrated that our novel ligands are able to visualize PMSA-positive tumors using both radionuclide and fluorescence imaging in a mouse model. The new backbone composition significantly improved tumor targeting in the PSMA-positive xenograft model compared to PSMA-N064 (*P* < 0.01). Although a direct comparison is difficult due to differences in measurement time points and tumor model used, tumor uptake values of radiolabeled tracers such as PSMA-617, PSMA-I&T, PSMA-1007, and PSMA-I&F reported in the literature range from 5 to 13%ID/g (LNCaP, 1/2 h p.i) [10, 33–35], whereas the uptake of PSMA-N01, -N02, and N03 in the current study was ≥ 20%ID/g (LS174T-PSMA, 2 h p.i.). In addition, a previous direct comparison of the LNCaP and LS174T-PSMA xenograft models did not show major differences in PSMA-I&T tracer uptake between these models [35], indicating that the performance of PSMA-N01, -N02, and N03 is in a similar range to those of the clinically available ligands.

As expected, we measured very low uptake in PSMA-positive tumors when using our control ligand PSMA-N064inc, signifying the PSMA specificity of the ligands and the need for an intact PSMA binding motif. Withal, these findings support the increasing evidence that properties such as charge, ability to fit the entrance funnel of PSMA, and overall molecular structure of the ligands contribute to efficient in vivo tumor targeting.

Although not dual-labeled, two IRDye700DX-based PSMA ligands suited for PDT have been reported in the literature with IC_50_ values in the low nanomolar range similar to our ligands [12, 36]. However, in the study of Wang et al. [36], in vitro incubation of PC3-pip cells with 1-µM IRDye700DX-labeled PSMA ligand and subsequent NIR light exposure did not lead to any PDT effects, whereas in our study, incubation with 30-nM dual-labeled ligand led to a significant decrease in cell viability. Interestingly, in vivo PDT subsequent to fluorescence-guided surgery using a IRDye700DX-conjugated PSMA ligand was shown to reduce tumor recurrence and significantly elongate animal survival compared with white light surgery [37]. The preclinical feasibility of multimodal intraoperative image guidance with subsequent ablative PSMA-targeted PDT using a dual-labeled tracer was first shown using the murine antibody [^111^In]In-DTPA-D2B-IRDye700DX [9]. In recent literature, a first dual-labeled photosensitizer-based PSMA ligand was described named LC-pyro, a PSMA ligand coupled to a porphyrin photosensitizer that can be labeled with copper-64 (^64^Cu) for PET imaging. However, the positron emitter ^64^Cu is difficult to detect with a gamma probe system during surgery and therefore not suitable for radio-guided surgery [38].

In conclusion, we modified the PSMA peptide linker by substitution of a glutamic acid into a lysine residue, providing a handle for conjugation of multiple functional moieties. Using this new backbone, we synthesized and characterized three dual-labeled ligands for intraoperative radiodetection, fluorescence-guided surgery, and PDT of PCa. Ligand modification performed in our study showed that the new backbone structure (PSMA-N01, -N02, and -N03) leads to high-affinity dual-labeled PSMA ligands with excellent PSMA-specific tumor uptake. These results encourage further preclinical and clinical testing of the dual-labeled ligands to refine the surgical treatment of PCa.

## Supplementary Information

Below is the link to the electronic supplementary material.

Supplementary file1 (DOCX 2193 KB)

## Abbreviations

^111^In: Indium-111
NIR: Near infrared
PCa: Prostate cancer
PSMA: Prostate-specific membrane antigen
PDT: Photodynamic therapy
