# Supplementary material for: Theranostic PSMA ligands with optimized backbones for intraoperative multimodal imaging and photodynamic therapy of prostate cancer
Source: Eur J Nucl Med Mol Imaging. 2022 Jan 14;49(7):2425–35. doi: 10.1007/s00259-022-05685-0 (PMC9165289; doi:10.1007/s00259-022-05685-0)
Supplement: Supplementary file 1 — Supplementary file1 (DOCX 2193 KB) [file 259_2022_5685_MOESM1_ESM.docx]

**Supplementals**

| 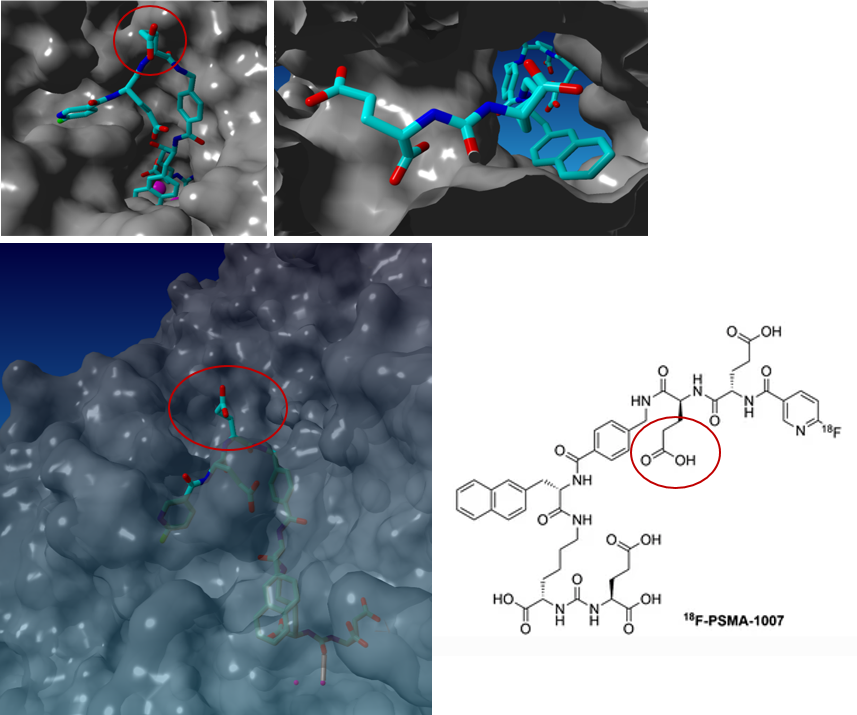 |
| --- |
| **Supplementary figure 1. Crystal structures of PSMA-1007 in the active site of PSMA.** Top left: pockets in the entrance funnel of PSMA are occupied by naphtylalanine, aminomethyl benzoic acid, two glutamates and fluorinated nicotinic acid of PSMA-1007. Top right: PSMA binding motif Glu-urea-Lys in the active site of PSMA. Bottom left: View of the entrance of the PSMA binding pocket with the C-terminal glutamic acid side chain sticking out (red circle). Bottom right: Structure of PSMA-1007 with C-terminal glutamic acid circled in red. Surface of PSMA is indicated in grey. Oxygen atoms (red), nitrogen atoms (dark blue) and fluorine atom (green) of PSMA-1007 (bonds, light blue) are indicated, as well as active site Zn (purple). PDB file 5O5T (*1-3*). |

**Supplementary Materials and Methods**

*DIPCDI coupling of protected amino acids:*

Fmoc-protected amino acid (3.0 eq.), 1- hydroxybenzotriazole hydrate (HOBt, 1M in DMF, 3.6 eq.), N, N’-Diisopropylcarbodiimide (DIPCDI, 1M in DMF, 3.3 eq.) were added to the resin and agitated until the Kaiser test was negative (~45 minutes) after which the resin was capped with a mixture of Ac_2_O (10 eq) and pyridine (10 eq) in DMF for 5 minutes and subsequently washed with DMF (3x10 mL).

*HATU coupling of protected amino acids*:

Fmoc-protected amino acid (3.0 eq.), 1- hydroxybenztriazole hydrate (HOBt, 3.6 eq.), N, N, N′, N′-Tetramethyl-O-(1H-benzotriazol-1-yl)uronium hexafluorophosphate (HBTU, 2.9 eq.) and N, N’-diisopropylethylamine (DIPEA, 6 eq.) were dissolved in DMF. The solution was pre-activated for 2 minutes before it was added to the resin. The mixture was agitated until the Kaiser test was negative (~1.5 hrs.) after which the resin was washed with DMF (3x10 mL) and DCM (3x10 mL).

*Fmoc removal:*

The resin was treated with 20% piperidine in DMF 3x6 minutes. The product was washed with DMF (3x10 mL).

*Alloc removal:*

Phenylsilane (25eq) and tetrakis(triphenylphosphine)palladium(0) (0.3eq) in DCM were added to the resin. The mixture was agitated for 20-30 minutes under a stream of argon (upon which the color changed from yellow to dark brown). Next the resin was washed with DCM, DMF and sodium diethyldithiocarbamate (0.5% in DMF) until the brown color had completely disappeared (generally 3 times).

**Supplementary figure 2**. **Synthesis of PSMA-N01, N02 and N03.**

*Synthesis of* PSMA-N01, -N02 and -N03

i) Wang resin (1 eq., 1.0 mmol/g, 1.00 g) was swollen in 10 mL DMF for 10 minutes. Fmoc-Lys(Alloc)-OH (3 eq., 3 mmol, 1.87 g), 4-dimethylaminopyridine (1 eq., 1 mmol, 122.2 mg), HOBt (3.6 eq., 3.6 mmol, 1M in DMF) and DIPCDI (3.3 eq., 3.3 mmol, 1M in DMF) were added to the resin and mixed on a bench roller for 20 hours. The reagents were removed from the resin by vacuum filtration. The resin was washed with DMF (3x10 mL) and DCM (3x10 mL). The Fmoc-loading was determined to be 0.5 mmol/g. Next, the resin was capped with a solution of pyridine (0.34 mL/g resin) and benzoyl chloride (0.34 mL/g resin) in DCM for 1 hour.

ii) The resin was washed with DCM (3x10 mL) and DMF (3x10 mL) and after Fmoc removal DIPEA (0.52 mL, 3 eq., 3 mmol,), 4-nitrophenyl chloroformate (2 eq., 2.0 mmol, 402 mg) in 2 mL DCM were added to the H-Lys(Mtt)-resin (1eq, 0.5 mmol/g, 2 g) and the resin was agitated for 1 hour. Consecutively a Kaiser test was performed to check for completion (*4*).

iii) Glutamic acid di-*tert*-butyl ester hydrochloride (3 eq., 3 mmol, 887.4 mg) and DIPEA (4 eq., 4 mmol, 0.70 mL) in DCM were added to the resin and the mixture was agitated for 1 hour. The resin was washed with DCM (3x10 mL) and DMF (3x10 mL).

iv) After Alloc removal, Fmoc-3-(2-naphthyl)-L-alanine (Fmoc-Nal) was coupled.

v) After Fmoc removal either Fmoc-(4-aminomethyl)benzoic acid (Fmoc-Amb) or trans-4-(amino-methyl)cyclohexane-1-carboxylic acid (Fmoc-Amc) was coupled.

vi) After Fmoc removal Fmoc-Lys(Alloc)-OH was coupled

vii) After Fmoc removal Fmoc-Glu(OtBu)-OH was coupled

viii) After Fmoc removal benzoic acid or nicotinic acid was coupled using the peptide coupling protocol but using HBTU instead of HATU.

ix) After Alloc removal, Fmoc-Lys(Alloc) was coupled.

x) DIPEA (2 eq.) and DOTA-OSu were added to the resin in NMP and mixed on a bench roller at room temperature for 6-8 hrs. Upon a negative Kaiser test the resin was washed with DMF (3x), DCM (3x), MeOH (3x) and diethyl ether (3x).

xi) The peptide was cleaved from the resin with trifluoroacetic acid/H_2_O (95:5, v/v) for two hours after which the resin was filtered off and the peptide was precipitated in diethyl ether. After drying in air the crude peptide was lyophilized from water and purified with preparative RP-HPLC.

xii) To 1 equivalent of compound **11a-c** in phosphate buffer pH 8, 700DX-OSu ester in DMF was added and stirred at room temperature for 4-5 hrs after which the product was purified directly with preparative RP-HPLC.

*Analytical an preparative HPLC*:

Analytical: Compounds were analyzed on a Shimadzu LC-20A Prominence system with a dual UV-Vis detector (Shimadzu, ‘s Hertogenbosch, The Netherlands) equipped with a C18 Gemini-NX column, 150 × 3 mm, particle size 3 μm (Phenomenex, Utrecht, The Netherlands) applying a gradient of 20-70% methanol in triethylammonium acetate buffer (10 mM, pH 7) for all IRDye containing compounds or a gradient of 5-100% acetonitrile in water (0.1% TFA) for all others.

Preparative: All compounds were purified on a Shimadzu dual-pump LC-20A Prominence system (Shimadzu, ‘s Hertogenbosch, The Netherlands) equipped with a C18 Gemini-NX column, 150 × 10 mm, particle size 10 μm (Phenomenex, Utrecht, The Netherlands), applying a gradient of 20-70% methanol in triethylammonium acetate buffer (10 mM, pH 7) for all IRDye700DX containing compounds or a gradient of 5-100% acetonitrile in water (0.1% TFA) for all others.

*PSMA-N064 blocking experiment:*

Five mice per group were intravenously injected with either 0.1 nmol PSMA-N064, 0.3 nmol PSMA-N064, 0.1 nmol PSMA-N064 + 10 nmol unlabeled PSMA-617 (100x excess) or PSMA-617. Ligands were labeled with 10 MBq ^111^In in PBS+0.5% (w/v) BSA. For the *ex vivo* biodistribution four groups (one for each experimental condition) of five mice were included. Two hours p.i., all mice from all groups were euthanized by CO_2_/O_2_-asphyxiation and relevant tissues were dissected, weighed and measured for radioactivity in a gamma-counter (2480 WIZARD^2^ Automatic Gamma Counter, PerkinElmer).

**Supplementary results**

| Table S1. Biodistribution of ^111^In-labeled ligands in mice bearing LS174T-PSMA and LS174T wildtype xenografts after dissection at 2 h p.i. Data expressed as %ID/g ± SD | | | | | |
| --- | --- | --- | --- | --- | --- |
|  | **PSMA-N01** | **PSMA-N02** | **PSMA-N03** | **PSMA-N064** | **PSMA-N064inc** |
| Biodistribution | | | | | |
| Blood | 1.1 ± 0.1 | 0.5 ± 0.01 | 0.7 ± 0.1 | 0.3 ± 0.1 | 0.3 ± 0.2 |
| Muscle | 0.2 ± 0.03 | 0.2 ± 0.04 | 0.2 ± 0.1 | 0.1 ± 0.04 | 0.1 ± 0.1 |
| Tumor- LS174T | 1.1 ± 0.2 | 0.6 ± 0.2 | 1.1 ± 0.2 | 0.7 ± 0.3 | 0.4 ± 0.1 |
| Tumor+ LS174T-PSMA | 21.2 ± 3.1 | 23.0 ± 2.0 | 19.8 ± 1.9 | 13.5 ± 2.4 | 0.5 ± 0.2 |
| Heart | 0.6 ± 0.1 | 0.4 ± 0.05 | 0.4 ± 0.04 | 0.4 ± 0.1 | 0.3 ± 0.1 |
| Lung | 2.7 ± 0.9 | 1.0 ± 0.3 | 1.8 ± 0.4 | 0.5 ± 0.1 | 0.3 ± 0.1 |
| Spleen | 2.2± 0.4 | 1.6 ± 0.2 | 1.5 ± 0.4 | 1.4 ± 0.3 | 0.2 ± 0.04 |
| Pancreas | 0.4 ± 0.1 | 0.3 ± 0.05 | 0.3 ± 0.02 | 0.3 ± 0.05 | 0.1 ± 0.04 |
| Liver | 1.5 ± 0.2 | 1.1 ± 0.1 | 1.3 ± 0.1 | 0.9 ± 0.2 | 0.6 ± 0.1 |
| Stomach | 0.7 ± 0.1 | 0.5 ± 0.1 | 0.6 ± 0.1 | 0.4 ± 0.1 | 0.4 ± 0.1 |
| Kidney | 99.2 ± 11.5 | 90.0 ± 6.6 | 142.4 ± 10.6 | 73.9 ± 17.4 | 47.3 ± 6.3 |
| Adrenals | 1.8 ± 0.1 | 1.2 ± 0.2 | 2.1 ± 0.8 | 1.6 ± 0.4 | 0.7 ± 0.6 |
| Duodenum | 0.7 ± 0.1 | 0.4 ± 0.05 | 0.4 ± 0.03 | 0.3 ± 0.1 | 0.2 ± 0.04 |
| Prostate | 0.6 ± 0.1 | 0.5 ± 0.1 | 0.6 ± 0.2 | 0.6 ± 0.1 | 0.6 ± 0.7 |
| Salivary glands | 0.8 ± 0.1 | 0.6 ± 0.1 | 0.6 ± 0.1 | 0.4 ± 0.04 | 0.3 ± 0.05 |
| Bone marrow | 0.3 ± 0.2 | 0.1 ± 0.1 | 0.2 ± 0.1 | 0.2 ± 0.1 | 0.1 ± 0.03 |
| Bone | 0.7 ± 0.1 | 0.5 ± 0.04 | 0.5 ± 0.1 | 0.7 ± 0.1 | 0.2 ± 0.1 |
| Tumor/Organ ratios | | | | | |
| Tumor/Blood | 20.0 ± 2.5 | 43.1 ± 4.1 | 27.0 ± 2.9 | 54.0 ± 14.7 | 1.5 ± 0.6 |
| Tumor/Kidney | 0.2 ± 0.04 | 0.3 ± 0.01 | 0.2 ± 0.02 | 0.2 ± 0.03 | 0.01 ± 0.003 |
| Tumor/Negative tumor | 19.7 ± 6.5 | 42.2 ± 16.4 | 19.9 ± 6.3 | 23.8 ± 9.7 | 1.1 ± 0.5 |
| Tumor/Spleen | 9.9 ± 1.4 | 14.3 ± 2.5 | 14.3 ± 4.0 | 9.6 ± 0.4 | 2.1 ± 0.5 |
| Tumor/Liver | 14.6 ± 2.4 | 21.6 ± 1.6 | 15.1 ± 1.7 | 15.6 ± 3.6 | 0.7 ± 0.2 |
| Tumor/Salivary gland | 27.8 ± 4.8 | 39.2 ± 3.7 | 35.0 ± 5.0 | 33.8 ± 6.6 | 1.6 ± 0.5 |
| Tumor/Prostate | 35.6 ± 4.6 | 46.9 ± 13.8 | 35.1 ± 10.7 | 21.9 ± 1.7 | 1.3 ± 0.9 |

| 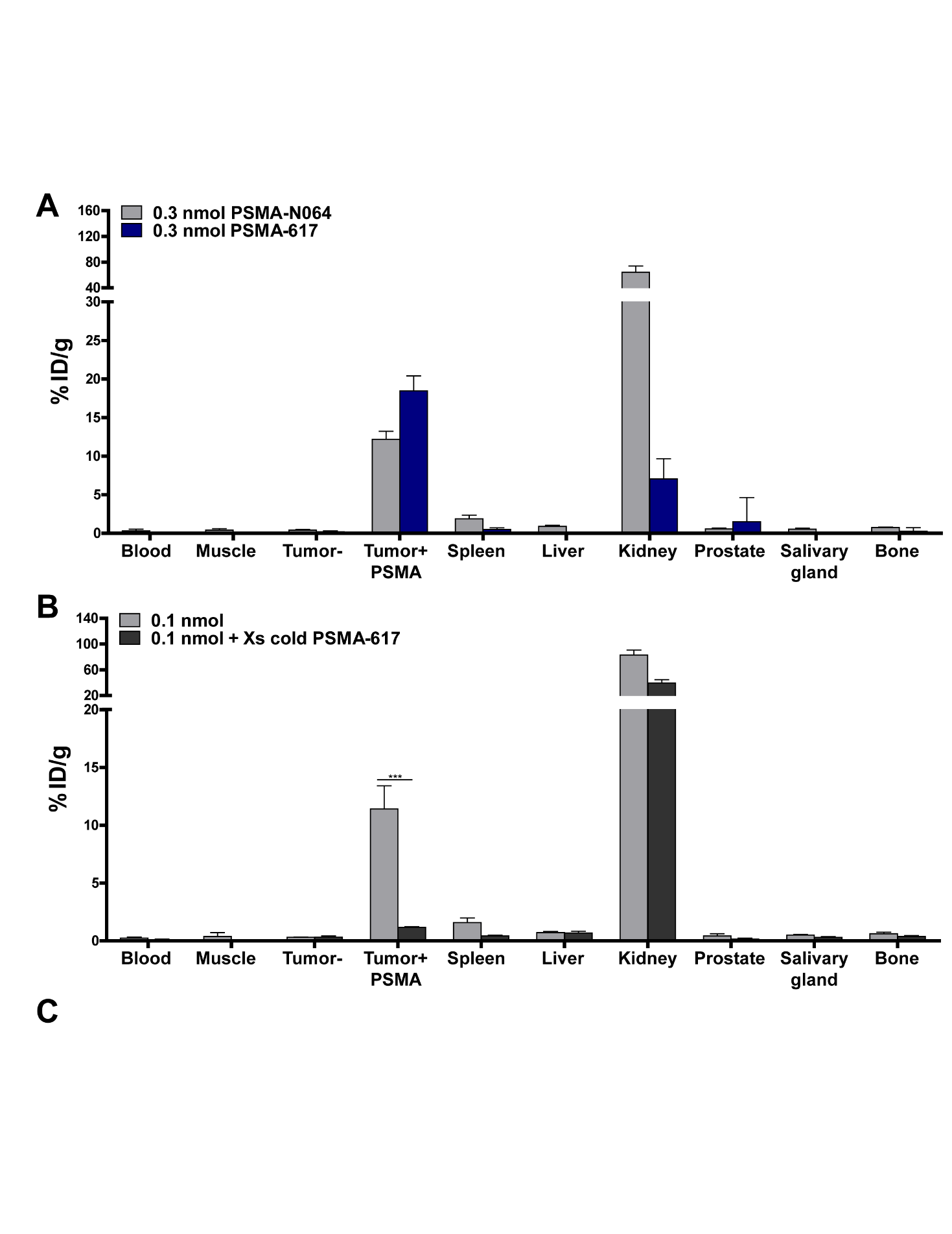 |
| --- |
| Supplementary figure 3. [^111^In]In-DOTAGA-PSMA-N064 blocking experiment.  Biodistribution as determined after dissection of ^111^In-labeled (A) PSMA-N064 (0.3 nmol/mouse) and positive control PSMA-617 (0.3 nmol) and (B) PSMA-N064 (0.1 nmol) and PSMA-N064 (0.1 nmol) + unlabeled PSMA-617 (block, 10 nmol). Biodistribution was determined 2 h p.i. (10 MBq/mouse, n= 5/group). Biodistribution was measured in mice bearing subcutaneous LS174T-PSMA (labeled Tumor+ PSMA) and LS174T wildtype (labeled Tumor-) xenografts. Data is expressed as %ID/g ± SD, ^**^ indicates P < 0.01, ^***^ indicates P < 0.001. |

**Table S2.** Biodistribution of ^111^In-labeled ligands in mice bearing LS174T-PSMA and LS174T wildtype xenografts after dissection at 2 h p.i. Data expressed as %ID/g ± SD

|  | PSMA-N064 (0.1 nmol) | PSMA-N064 (0.1 nmol) + PSMA-617 (10 nmol) | PSMA-N064 (0.3 nmol) | PSMA-617  (0.3 nmol) |
| --- | --- | --- | --- | --- |
| Biodistribution | | | | |
| Blood | 0.2 ± 0.2 | 0.2 ± 0.1 | 0.4 ± 0.3 | 0.1 ± 0.02 |
| Muscle | 0.4 ± 0.4 | 0.1 ± 0.1 | 0.5 ± 0.3 | 0.1 ± 0.1 |
| Tumor- LS174T | 0.4 ± 0.1 | 0.4 ± 0.2 | 0.5 ± 0.1 | 0.2 ± 0.2 |
| Tumor+ LS174T-PSMA | 11.5 ± 2 | 1.2 ± 0.1 | 12.2 ± 1.1 | 18.5 ± 1.9 |
| Spleen | 1.6 ± 0.4 | 0.5 ± 0.1 | 1.9 ± 0.5 | 0.5 ± 0.2 |
| Liver | 0.8 ± 0.1 | 0.7 ± 0.2 | 1 ± 0.2 | 0.1 ± 0.02 |
| Kidney | 82.9 ± 7.8 | 39.5 ± 5. | 64.4 ± 9.7 | 7.1 ± 2.6 |
| Prostate | 0.5 ± 0.2 | 0.2 ± 0.1 | 0.6 ± 0.2 | 1.5 ± 3.1 |
| Salivary glands | 0.6 ± 0.1 | 0.4 ± 0.1 | 0.6 ± 0.2 | 0.1 ± 0.02 |
| Bone | 0.7 ± 0.2 | 0.4 ± 0.1 | 0.8 ± 0.1 | 0.3 ± 0.5 |
|  |  | | | |

**Supplementary figure 4. HPLC and MALDI-ToF spectra of PSMA-N01, PSMA-N02, PSMA-N03, PSMA-N064 and PSMA-N064inc.**

***PSMA-N01***

HPLC chromatogram


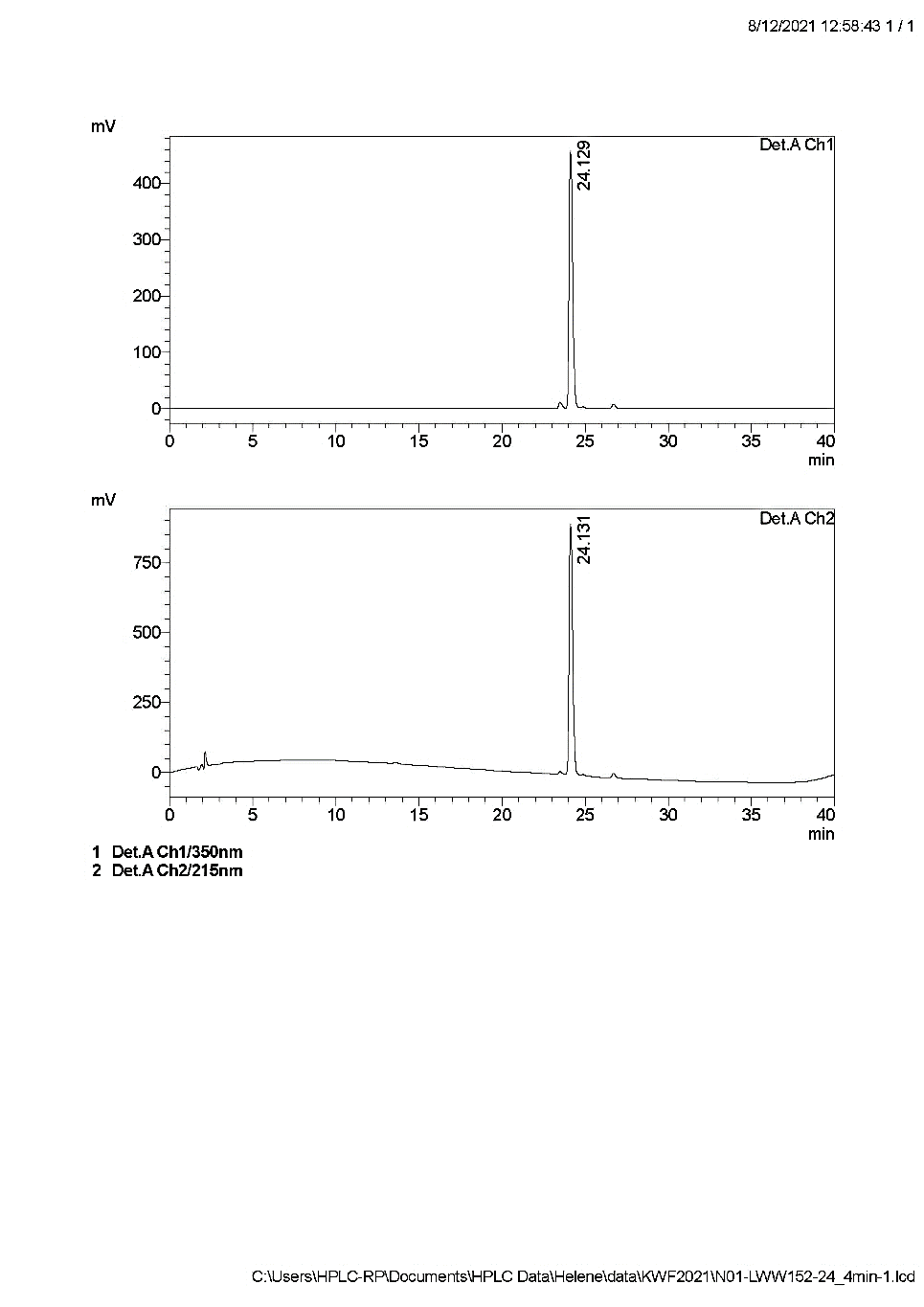


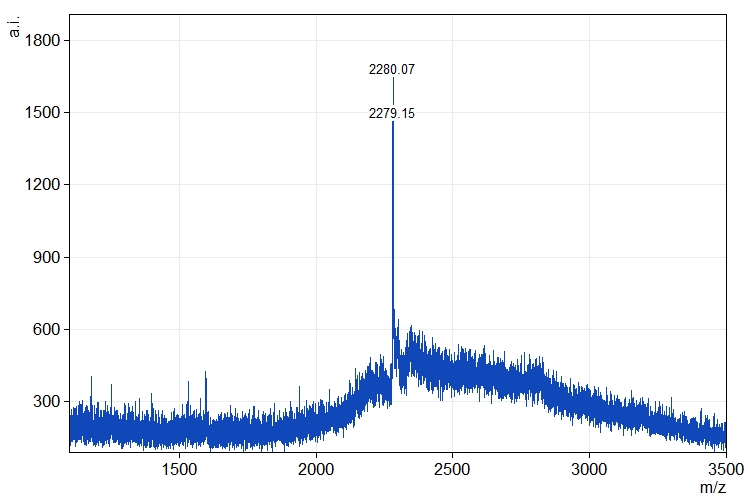
MALDI-ToF spectrum with matrix **HCCA**

***PSMA-N02***

HPLC chromatogram


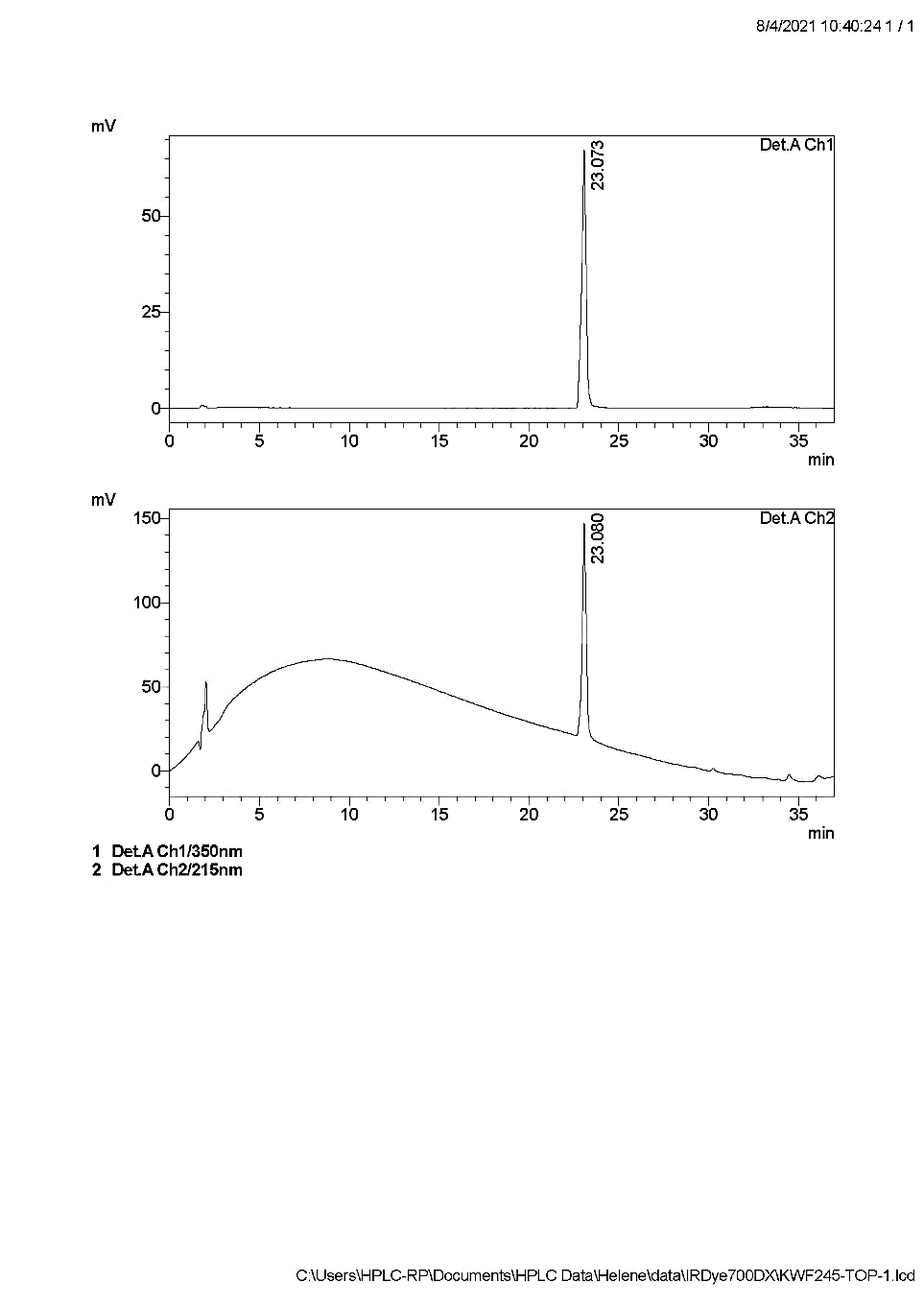


MALDI-ToF spectrum with matrix **HCCA**
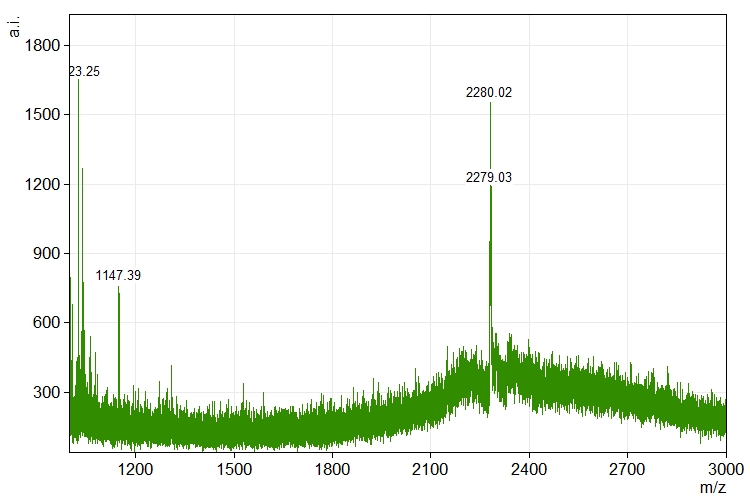

***PSMA-N03***

HPLC chromatogram


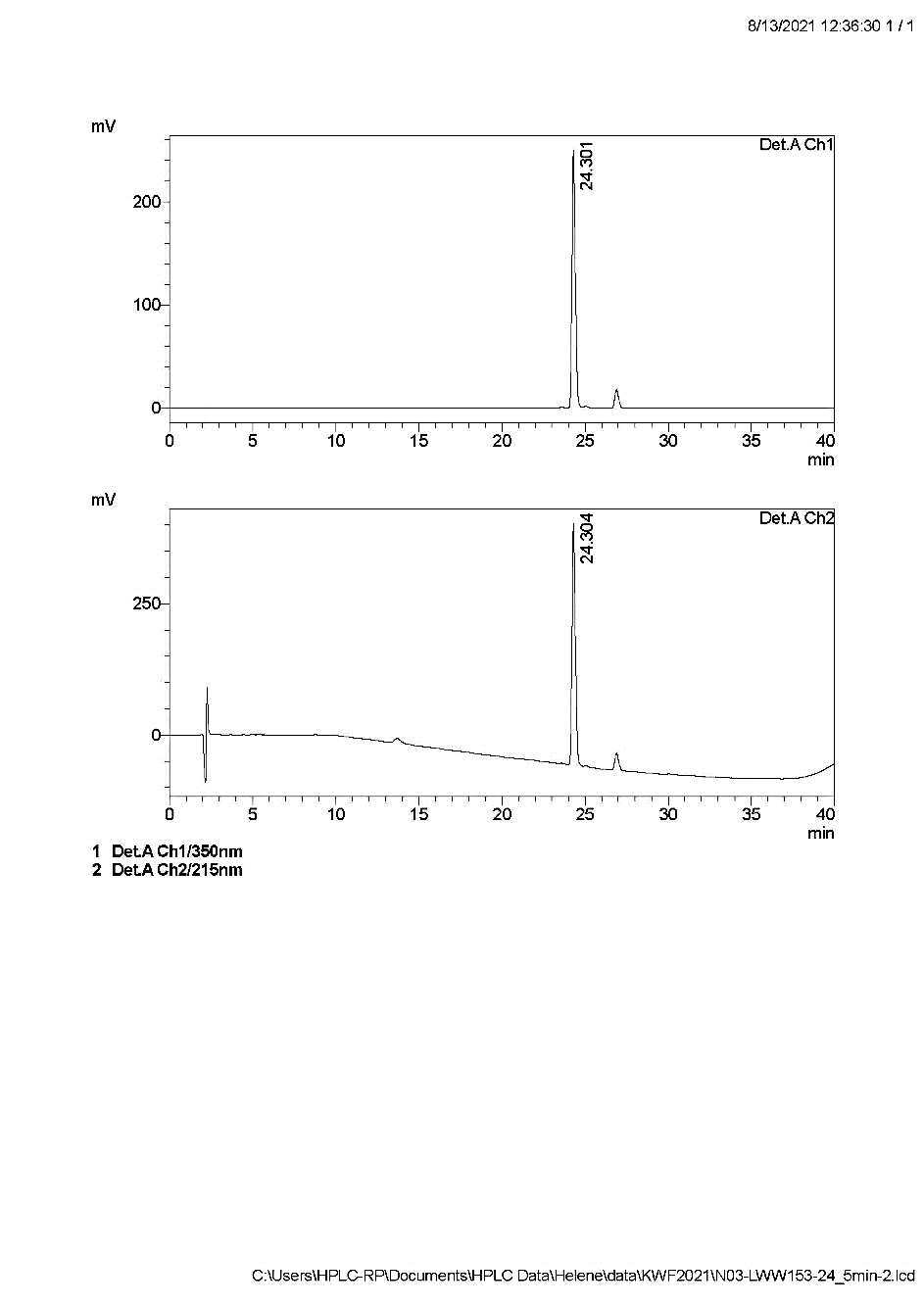


MALDI-ToF spectrum with matrix **HCCA**
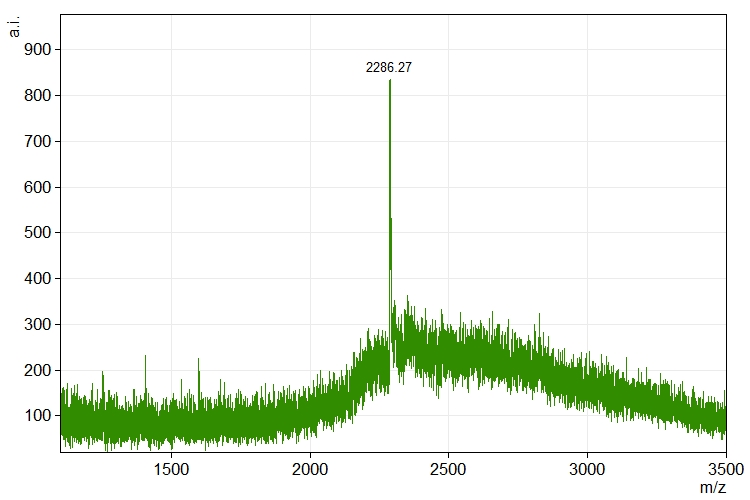

***PSMA-N064***

HPLC chromatogram

**
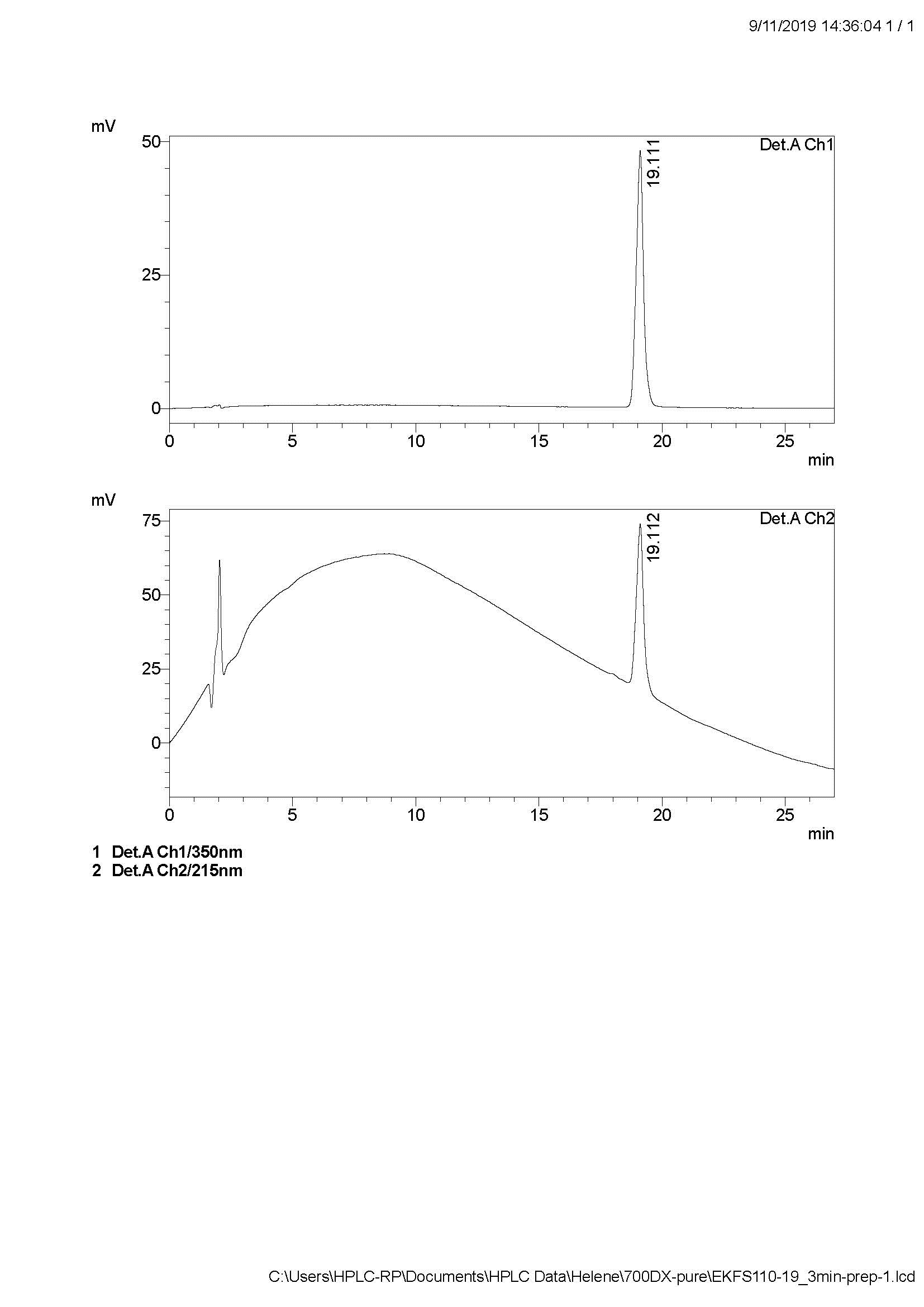
**

MALDI-ToF spectrum with matrix **HCCA**
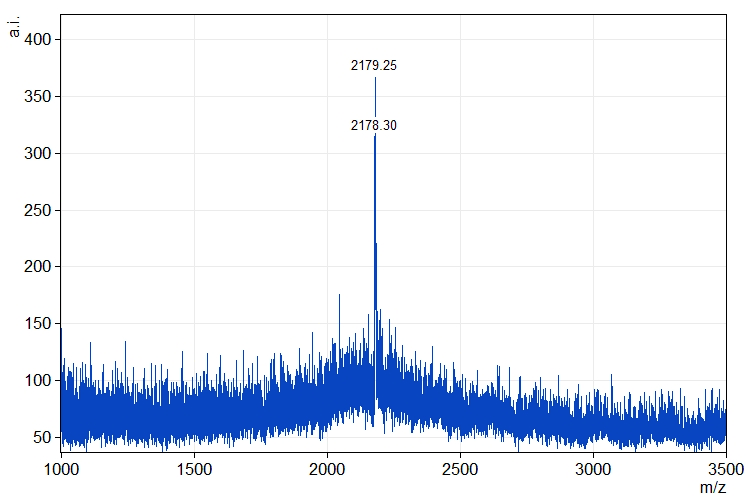

***PSMA-N064inc***

HPLC chromatogram


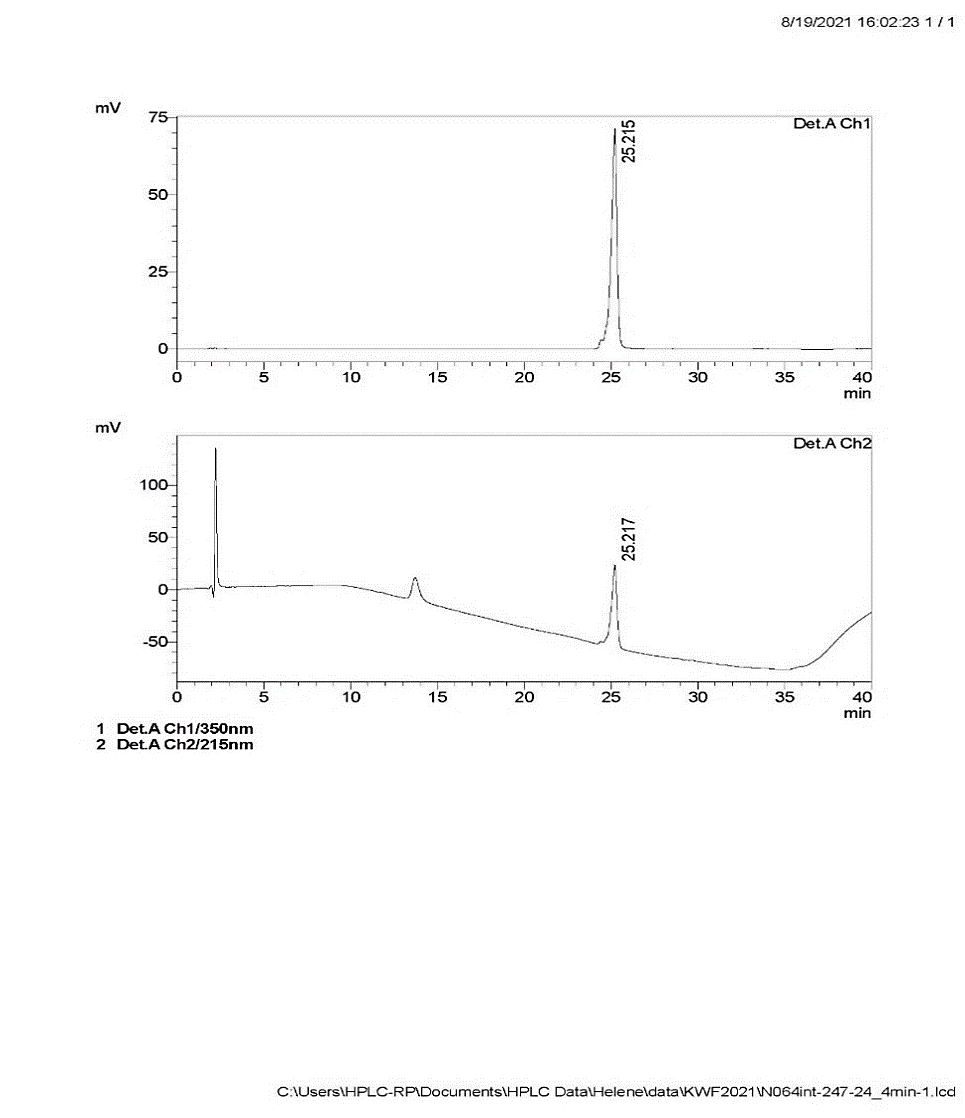


MALDI-ToF spectrum with matrix **HCCA
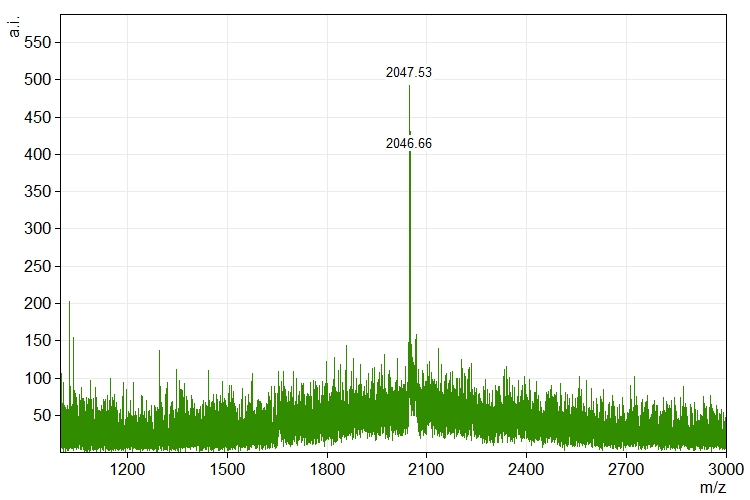
**

**References**

**1.** Cardinale J, Schäfer M, Benešová M, et al. Preclinical Evaluation of (18)F-PSMA-1007, a New Prostate-Specific Membrane Antigen Ligand for Prostate Cancer Imaging. *J Nucl Med.* 2017;58:425-431.

**2.** Barinka C, Byun Y, Dusich CL, et al. Interactions between human glutamate carboxypeptidase II and urea-based inhibitors: structural characterization. *J Med Chem.* 2008;51:7737-7743.

**3.** Barinka CN, Z.; Motlova, L. X-ray structure of human glutamate carboxypeptidase II (GCPII) in complex with a urea based inhibitor PSMA 1007. *Protein Data Bank* 2018;PDB ID: 505T

**4.** Kaiser E, Colescott RL, Bossinger CD, Cook PI. Color test for detection of free terminal amino groups in the solid-phase synthesis of peptides. *Anal Biochem.* 1970;34:595-598.
